# Postoperative nutritional support of the patient with gut gangrene—a case report

**DOI:** 10.1186/s41043-019-0169-1

**Published:** 2019-04-09

**Authors:** Samra Imran, Afifa Tanweer

**Affiliations:** 1 Department of Food and Nutrition, Government College of Home Economics, Lahore, Pakistan; 2 School of Health Sciences, University of Management and Technology, Lahore, Pakistan

**Keywords:** Mesenteric ischemia, Intestinal resection, Postoperative diet, Nutritional care

## Abstract

**Background:**

Bowel necrosis is a commonly observed condition in elderly patients with longstanding diabetes. In such condition, intestinal resection is usually performed for the removal of the gangrenous part. Post-surgical dietary management after bowel resection poses several challenges for the health care team.

**Case presentation:**

The case presented in this study is that of an elderly diabetic male who developed acute renal failure as a result of neglect in post-surgical feeding after intestinal resection. After the intervention by a trained dietitian, a transitional diet was planned and successfully executed, resulting in reversal of acute renal failure, dehydration, and post-surgical stress. Several complications including hepatic dysfunction and mouth ulcers were resolved through well-planned transitional diet. The patient was finally discharged in a stable health condition and was regularly followed up for any nutritional or medical issues.

**Conclusion:**

Neglects in nutritional care of patients can have severe implications including development of medical complications, resulting in increased length of hospital stay, augmenting the disease stress of the patient and family, and finally the preventable drainage of several human and monetary resources. Therefore, recognition of nutritional intervention as an important part of in-hospital health care may have social as well as economic impacts.

## Background

Mesenteric ischemia is a major cause of localized bowel necrosis for which surgical removal of the gangrenous part is the usual treatment procedure performed [1]. Post-resection management of the patient is, however, challenging for the health care facilitators, the caregivers, and even the patient himself. Although the remnant intestines undergo structural and functional adaptations as part of natural compensatory mechanism [2], short bowel syndrome, following the major intestinal resections, poses serious threats to nutritional, hydration, and overall health status of the patient [3].

In-hospital postoperative complications and readmissions have been reported to be quite common after intestinal resection [4]. Close monitoring and careful management of surgical patients is necessary to prevent the development of complications and to anticipate the need for readmission. The role of nutrition in improving the surgical patient’s health outcomes has lately been recognized; resulting in extraordinary progress in parenteral and enteral feeding associated with fewer post-surgical complications, decrease in the length of hospital stay, and favorable cost-benefit outcomes [5].

In developing regions of the world, the role of dietitians as part of health care team is well under-recognized. In Pakistan as well, nutrition and dietetics are relatively newer fields with rare representation of dietitians, especially in the public sector hospital setups. In this era of preventive medicine and sustainable development, the economic and social benefits that can be gained through close nutritional monitoring of patients warrant great advances, especially in the developing world. The case presented in this report highlights the significance of appropriate and timely nutritional intervention in preventing and reversing surgical complications most likely to occur after intestinal resection.

## Case presentation

A 70-year-old type II diabetic male with the history of gut gangrene presented at surgical department of a public sector tertiary care hospital in Lahore, Pakistan. He went through surgical procedure of partial small intestinal resection, sparing 1 ft proximal jejunum and 2–3 ft distal ileum. Drainage jejunostomy was performed. After the surgery, the patient was kept on inadequate peripheral parenteral nutrition (PPN) (2 l/day) for 4 days. Excessive losses through the fistula during this period and unnoticed hydration inadequacy led to the development of hypovolemia. Based upon biochemical analysis, elevated BUN (115 mg/dl), and creatinine levels (7.2 mg/dl), the patient was diagnosed with acute renal failure. The random blood glucose levels were found to be within controlled range (148 mg/dl). He was referred to nephrology department of the same health care facility where hypovolemia was reversed through the administration of intravenous rehydration fluids (saline 5% dextrose solution with KCl 2.5 cc/ml). After 15 days, acute renal failure was settled; BUN and creatinine reached within normal range (16 mg/dl and 1.1 mg/dl, respectively). However, the patient on discharge was given an inappropriate ileostomy feed plan including ORS (800 ml/day), milk (600 ml/day), polymeric supplemental formula (1800 ml/day), and low dose aspirin therapy through feeding ileostomy. Moreover, he was given broth and blended bread slices by his caregivers through ileostomy. Poorly planned ileostomy feed led to several complications, resulting in increased hospital stay, and serious implications on his physical, psychological, and social wellbeing.

After 23 days, the patient returned to the hospital with relapse of acute renal failure (BUN = 32 mg/dl, creatinine = 1.6 mg/dl). The patient showed signs of severe dehydration along with pedal edema upon physical examination. A history of 16% weight loss within the last month (BMI = 19.5 kg/m^2^), mild anemia (Hb = 11.7 g/100 ml), and hypoalbuminemia (2.8 g/dl) indicates poor nutrient uptake and declining nutritional status. Because of the declining health of the patient, early anastomosis was carried out to restore intestinal continuity. At this point, a trained dietitian intervened and planned a post-surgical progressive diet along with careful monitoring of hydration status. Total parenteral nutrition [TPN] was recommended initially with a gradual transition to oral feed. The target caloric requirement of the patient was calculated to be 2400 kcal with fluid intake of 6000–7000 ml.

For the first 2 days post-surgery, electrolyte and fluid balance were maintained through intravenous solutions. From the 3rd day onwards, an individualized TPN plan along with the administration of rehydration fluids through feeding ileostomy was started with the target of improving the nutritional status, allowing for postoperative stress and maintaining the hydration and electrolyte status (Table 1).

**Table 1:** Post-anastomosis total parenteral nutrition plan

Component	Amount fed (ml)	Vitamins	Electrolytes (mg)				Macronutrients (g)			Fluids (ml)	Kcal
			Na	K	Cl	Mg	CHO	Pro	Fat		
TPN solution											
25% dextrose	500	–	–	–	–	–	125	–	–	500	425
Sorbitol	150 g	–					150			0	
Amino acid solution	1500	–	1207	1462	2107	91.12	150 (sorbitol)	75	–	1500	900
20% fat emulsion (alternate days)	20 g	–	–	–	–	–	–	–	20	100	180
Injection Multibionta	10 ampule	Vitamin A (1000 IU), E (5 mg), C (500 mg), B1 (50 mg), B2 (10 mg), B3 (100 mg), B6 (10 mg)	–	–	–	–	–	–	–		–
Total										2100	1505

The caloric requirement of the patient for initial total parenteral feed was calculated to be 1700 kcal (BEE + 20% sedentary factor + 15% for uncomplicated surgery + 15% for severe weight loss). Initial TPN plan provided 1500 kcal from elemental formula (amino acids), sorbitol, and 25% dextrose solution. An intravenous fat emulsion (20% Liposyn) was infused on alternate days providing 20 g fat/day for preventing the fatty acid deficiency. In addition, a 10 ml ampoule of multivitamin (Multibionta) was administered daily in order to fulfill the requirements of vitamins A, E, C, and B complex (Table 1).

TPN was slowly tapered off by slow addition of ileostomy feed (Table 2) and then to final transition towards oral feed (Table 3).

**Table 2:** Post-anastomosis total parenteral nutrition and ileostomy plan

Component	Amount fed (ml)	Vitamins	Electrolytes (mg)				Macronutrients (g)			Kcal
			Na	K	Cl	Mg	CHO	Pro	Fat	
TPN solution										
Dextrose	1000	–	–	–	–	–	250	–	–	850
Aminovel	2000	–	1610	1950	2690	121.5	200 (sorbitol)	100	–	1200
Injection Multibionta	10 ampule	Vitamin A (1000 IU), E (5 mg), C (500 mg), B1 (50 mg), B2 (10 mg), B3 (100 mg), B6 (10 mg)	–	–	–	–	–	–	–	–
Total	–	Vitamin A (1000 IU), E (5 mg), C (500 mg), B1 (50 mg), B2 (10 mg), B3 (100 mg), B6 (10 mg)	1610	1950	2690	121.5	450	100	0	2050
Ileostomy feed										
ORS	1000	–	2850	750	–	–	20	0	0	68
Partially hydrolyzed supplement	100	–	56	140	–	–	18.5	4	1	100
Normal saline	3000	–	0	0	–	–	0	0	0	0
Total	4100	–	2906	890	–	–	38.5	4	1	168
Grand total	–	Vitamin A (1000 IU), E (5 mg), C (500 mg), B1 (50 mg), B2 (10 mg), B3 (100 mg), B6 (10 mg)	4516	2840	2690	121.5	488.5	104	1	2218

**Table 3:** Post-anastomosis progressive feeding plans

Type of diet	Diet constituent	Amount	Calories (kcal)
Clear fluid diet + TPN			
Clear fluid diet	Clear broth	500 ml (100 ml/3 h)	1025
TPN	Aminovel	1000 ml	
	25% dextrose	500 ml	
Full fluid diet+ TPN			
Full fluid diet	Strained porridge	½ C	952
	Chicken soup	½ C	
	Clear broth	1 C	
	Chicken soup	½ C	
TPN	Aminovel	500 ml	
	25% dextrose	500 ml	
Soft diet + PPN^a^			
Soft diet	Soft boiled egg	1	870
	Strained porridge	1 C	
	Chicken soup	½ C	
	Rice (*khichri*)	3–4 tbsp	
	Yogurt	50 g	
	Clear broth	1 C	
	Chicken soup	½ C	
	Rice (*khichri*)	3–4 tbsp	
	Yogurt	50 g	
PPN	10% dextrose	1000 ml	

^a^TPN was slowly tapered (due to raised LFTs) resulting in a slight dip in total caloric intake which was gradually recovered by the addition of polymeric supplement and banana

A high protein, high carbohydrate (low lactose, low fiber), and low-fat diet was planned (Table 3). On the 5th day after anastomosis, the patient received first oral intake of clear fluid diet along with TPN. The total calories were kept within the range of 1100–1500 kcal. Upon introduction of oral feed, severe dysphagia and withdrawal of food were observed which, upon probing, was revealed to be due to the overlooked oral hygiene and prolonged NPO period with subsequent pharyngitis and mouth ulcers. Antiseptic mouthwash was started promptly. The patient’s caregivers were counseled regarding effective feeding and caring practices. The patient also contracted urinary tract infection for which antibiotics were prescribed. Mobility was encouraged by suggesting supported sitting and walking along with frequent side changing while lying in order to recover from bed sores. After maintaining the tolerance of clear fluid diet for 2 days, the patient was shifted to full fluid diet along with TPN. Oral fluid intake (drinking water) was also started. On the next day, the patient was suspected to develop TPN-induced hepatic dysfunction based on clinical signs of jaundice which was confirmed by the abnormal pattern of hepatic enzymes (ALP = 465 U/L, ALT = 40 U/l, AST = 52 U/l) and hypoalbuminemia. TPN was discontinued.

Soft, bland diet (Table 3) consisting of easily digestible food items from all food groups and nutritional supplement along with PPN (10% dextrose), intravenous albumin infusion, and oral multivitamin syrups was then introduced. Soft diet was continued for 2 weeks with gradual increase in calories. The patient was discharged with a recommendation of a detailed soft, bland diet plan able to meet 1750 Kcal (Table 4).

**Table 4:** Diet plan recommended on hospital discharge

Meal time	Foods recommended	Amount	Exchanges	CHO (g)	Pro (g)	Fats (g)	TAG (g)	Kcal
Breakfast	Soft boiled egg	1	1 medium fat meat	–	7	5	4	75
	Chicken soup (added corn flour)	250 ml	1 lean meat	–	7	3	3.8	55
		1 tbsp corn flour	1/3 starch	5	1	0	5.5	26.7
Midmorning	Yogurt	125 ml	0.8 whole milk	9.6	6.4	6.4	13.44	120
	Apple sauce	1 apple	1 fruit	15	–	–	15	60
Lunch	Chicken soup (added corn flour)	250 ml	1 lean meat	–	7	3	3.8	55
		1 tbsp corn flour	1/3 starch	5	1	0	5.5	26.7
	Rice (*khichri*)	1 C (total)	2 starch	30	6	2	33.2	160
	Lentil	1½ tbsp.	1 very lean meat	–	7	0	3.5	35
	Yogurt	50 ml	0.27 whole milk	3.24	2.16	2.16	4.536	40.5
Tea time	Polymeric supplement	250 ml	–	38.26	7.14	13.64	43.194	304.36
	Rusks/crackers	2	1 starch	15	3	1	16.6	80
Dinner	Chicken soup (added corn flour)	250 ml	1 lean meat	–	7	3	3.8	55
		1 tbsp corn flour	1/3 starch	5	1	0	5.5	26.7
	Rice (*khichri*) with added Lentil	1 C (total)	2 starch	30	6	2	33.2	160
		1 ½ tbsp.	1 very lean meat	–	7	0	3.5	35
	Banana	½	1 fruit	15	–	–	15	60
	Yogurt	50 ml	0.27 whole milk	3.24	2.16	2.16	4.536	40.5
Bedtime	Polymeric supplement	250 ml		26	10	9	31.9	225
	Rusks/crackers	2	1 starch	15	3	1	16.6	80
Between meals	Water	1000 ml	–	–	–	–	–	–
Total				215.34	90.86	53.36	265.7	1666.01

The patient’s caretakers were guided for food substitutes for adding variety in diet and increasing the palatability and acceptance. The caregivers were guided for foods to avoid and were given balanced diet recipes for incorporating all the food groups in the diet (Table 5).

**Table 5:** Practical instructions for increasing dietary compliance of the patient upon discharge

Adding variety	- Lentil soup (1 ½ tbsp raw lentil) with chicken rice (1/2 pcs.) - Chicken with noodles instead of rice (½ C cooked noodles = 1/3 C rice) - Add noodles to chicken soup to reduce amount of *khichri* - Rice can be added in chicken soup
Substituting fruits	1 fruit means small apple = ½ banana = 1 small pear = 1 small peach = 10 small grapes = 2 plums = 1 slice water melon = ½ small mango = ½ C fruit cocktail/chaat
Addition of vegetables	Gradually add vegetables to rice and soup along with chicken to add variety
Foods not allowed	Containing high oxalate contents (spinach, okra, tea, whole grain wheat)

Throughout the nutritional management, the blood glucose levels of the patient were carefully monitored (below 180 mg/dl) and insulin units were precisely adjusted for the grams of carbohydrates fed. Two weeks after discharge, the patient was called for a follow-up. He showed compliance with dietary recommendations and medical prescription, and his health showed a gradual improvement. The patient was suggested to decrease the amount of nutritional supplement while increasing the exchanges for other foods. Dietary recommendation of adding 2 teaspoons of fat (canola oil) and chicken/vegetable curry was given. Local flat bread (chapatti) made of extracted wheat flour was allowed. On the next follow-up visit, the patient showed slight weight loss and reduced compliance to diet. The dosage of dietary supplement was again increased, and caregivers were given strict instructions for dietary compliance until the patient regained weight and gradually started having family meals with slight modifications.

## Discussion

Bowel necrosis and gangrene are among the devastating consequences of ischemic insult affecting bowel viability. Although it may occur at any age, the risks have been found higher in the elderly [6]. In addition to its risk being increased with age, the prevalence of GI complications has also been reported to be higher in diabetic patients compared with non-diabetics, primarily as a result of diabetic autonomic neuropathy involving the GI tract [7]. The study at hand is the case of an elderly, male, diabetic patient who presented with gut gangrene and subsequently underwent intestinal resection.

The case presented in this study demonstrated dehydration as the underlying cause of post-surgical complication and subsequent hospital readmission of the patient. Acute renal failure occurred due to negligence in calculating fluid losses through discharge from jejunostomy. Several complications have been reported in patients with mesenteric ischemia among which renal failure and dehydration are the most commonly occurring issues reported in more than half of the patients [8]. A multi-national epidemiological study on acute renal injury found a 50% prevalence of acute renal injury in critically ill patients. Mortality has been found to be associated with the severity of renal disease for ICU patients [9].

Mesenteric ischemia, especially after the development of infarction, remains a life-threatening condition with a mortality rate of 60–80%. Early diagnosis along with aggressive therapeutic approach is essential for preventing and improving the outcomes in such patients. Fluid resuscitation is a major challenge in the management of patients who have undergone surgical intervention for mesenteric infarction [6]. Readmissions rate after intestinal resection has previously been studied to be quite high, with dehydration being the major cause [10].

Even small acute changes in kidney function can result in short-term and long-term complications, including chronic kidney disease, end-stage renal disease, and death [11]. A slight misguidance in feeding plan of such patients can lead to severe complications. In the current study, the patient was recommended a polymeric high caloric formula through ileostomy which resulted in his readmission with relapse of acute renal failure and decline in nutritional status. Inclusion of polymeric formula instead of elemental formula in postoperative diet for jejunocolic anastomosis has been linked to negative clinical outcomes including diarrhea and longer duration of parental support [12].

Growing clinical evidence points to a high incidence of severe liver disease in chronic TPN-dependent patients [13]. In the current study, during the follow-up, the patient developed TPN-dependent liver dysfunction demonstrated as jaundice. Careful monitoring of patients is warranted in these circumstances. Close monitoring of the patient resulted in reversal of this condition.

In addition to the renal and hepatic complications, background disease processes, medication, and therapies in people with intestinal failure receiving home parenteral nutrition may also affect their oral health. Poor oral health has been reported in patients receiving parenteral nutrition compared with the general population [14]. Parenteral nutrition with unnoticed oral hygiene has been found to promote negative consequences like poor dentition and gingivae [15]. The present case study also indicated overlooked oral hygiene which created difficulty for the patient to take oral feed. Such underlying issues for failure to dietary compliance must be given due attention during the management of post-surgical patients.

Most of the restricted diets postoperative are often unpalatable, thus contributing to anorexia [16]. In practical terms, the patient must be provided with alternatives and substitutes for keeping into account the nutrition and psychological requirements of the patient and keeping in mind his preferences for food, individual diets must be recommended. Therefore, when substitutes were provided in the soft diet (Table 4 and Table 5) and detailed nutritional counseling of the patient and his caregivers was done, compliance consequently health status improved.

## Conclusion

In conclusion, minor neglects in care of patients may implicate economically, socially, and psychologically. Intervention by a trained dietitian for planning and executing feeding plans of in-hospital patients as well as a close follow-up may result in fewer complications and may significantly decrease the length of hospital stay. Modern-day patient care practice requires searching for underlying issues associated with non-compliance and providing practical approach to handle those issues. The promotion of nutritional care and counseling in hospitals can, therefore, play highly essential part for improving the health care system and in reducing the burden of disease complications in patients.
